# Tracing teachers’ perceptions of entanglement of digitally-mediated educational activities and learning environments: a practice-oriented method

**DOI:** 10.1007/s10984-022-09442-w

**Published:** 2022-11-24

**Authors:** Katri Sarkio, Tiina Korhonen, Kai Hakkarainen

**Affiliations:** Faculty of Educational Sciences, University of Helsinki, Helsinki, Finland

**Keywords:** Digital technology, Innovative learning environment, Methodological framework, Research-practice partnership, Sociomateriality

## Abstract

School spaces, technologies, and educational activities shape each other reciprocally. This mixed-methods study (i) developed a methodological framework for tracing the interdependence between school spaces, digital instruments, and teachers’ practices in their use, and (ii) created practice-based knowledge about the relations of teachers’ perceptions of educational activities to school spaces in order to acknowledge architects and educational practitioners in designing learning environments that support educational renewal. We participated in the construction process of a new general upper-secondary school building in Finland, taking into consideration the school’s operating culture and teachers’ pedagogic needs (social aspects), as well as the mediation of school spaces by digitalization and national curriculum reformation (material aspects). The data were collected through a teacher survey (*n* = 31), teacher interviews (*n* = 10), and the analysis of group notes that teachers (*n* = 39) generated in a participatory workshop. The results revealed that teachers consider it critical in the design of new learning environments to foster a collaborative and multidisciplinary school culture that emphasizes learners’ wellbeing and inclusion, as well as utilizing readily-available digital instruments with basic-level functionality. The developed methodological framework appeared suitable for visualizing (with radar charts) the sociomaterial interdependence of school spaces, digital instruments, and teachers’ personal and collective pedagogical activities. We argue that teachers’ perceptions of school spaces, digital instruments, and pedagogic practices entanglement consolidates learning environments design from an educational point of view and should be taken into consideration when constructing new school buildings.

## Introduction

The present article reports the findings of the first cycle of a design-based research project that traced teachers’ perceptions of digitally-infused school spaces and educational activities within the construction process of a new general upper-secondary school building in Finland. The new building is to represent the City of Helsinki’s vision of a sustainable future, in which creating safe and sound learning environments for children and adolescents is central in putting the real estate strategy in action (City of Helsinki, 2021). School buildings should be repaired or replaced with new buildings to meet the climate goals in terms of quality, schedule and finances, and carbon footprints. One solution in meeting these requirements that encompassed this investigation is a life span model. In the model, the City of Helsinki owns the forthcoming building, whereas a private construction company is responsible for the subsequent maintenance as a service provider several years after the construction, and for deployment.

Designing school buildings is an interdisciplinary process requiring reciprocal understanding between the participating stakeholders, including educators, architects, interior designers, and constructors (Tse et al., 2019). We aimed to contribute to the multifaceted participatory process by developing a methodological framework for tracing the interdependence between school spaces, digital instruments, and teachers’ practices and, thereby, to create practice-oriented knowledge for supporting the interdisciplinary design efforts of innovative learning environments (ILEs). ILEs are today’s disruptive interventions in the educational field (Imms, 2016; OECD [Organisation for Economic Co-operation and Development], 2013). Flexibility, diversity and increased openness characterize these contemporary learning environments, which have various designations, such as open, flexible, and innovative learning environments or spaces. We adopt the OECD’s (2013) term ILE, because it emphasizes learners’ individuality, sets collaboration central in promoting cross-curricular and inter-organizational activities, and accentuates the enormous reshaping potential of technologies. ILEs also aim to endorse the shift in educational activities from teacher to digitally-infused learner and learning-centricity. Learning-centricity, in Finnish general upper-secondary schools in the recently-introduced core curriculum (EDUFI, 2019) inter alia in cross-curricular activities, involves individual learning trajectories and educational practices that build on the learners’ knowledge and their active role. Additional to subject specific knowledge, the educational goals are to advance learners’ wellbeing, interaction, interdisciplinary, and creativity abilities as well as societal, ethical, environmental, global and cultural skills. These holistic skills and collaborative activities challenge not only subject-specific teaching traditions but also prevailing learning environments; closed classrooms and corridors with insufficient collaboration facilities and digital instruments dominate many schools. Therefore, the multidisciplinary stakeholders engaged in designing new learning environments need to share a common understanding of the pursued educational goals and practices in achieving those goals.

For the learning environments field, the visualized knowledge that the method creates serves as an input, on one hand, to the multidisciplinary stakeholders to design learning environments from the point of view of educational activities. The novel method exposes school spaces’ correspondence to educational needs from teachers’ perspective, which consolidates on the design of learning environments that are suitable for educational activities. That is to say, knowledge is created in visual and human interpretable format for input to the multidisciplinary stakeholders who collaborate in designing learning environments and need to understand the digitally-mediated socio-pedagogic activities occurring in school spaces. On the other hand, the created visualizations disclose cultivation needs in a school’s operational culture to transform toward a learning-centric collaborative school culture. The created knowledge is input to educational leaders to identify the needs and to direct support for those socio-pedagogic activities that should be strengthened in achieving the educational transformation process to take full benefit of ILEs when occupied. The entanglement of school architectures and educational activities has been given little attention in research, although knowledge and understanding of this phenomenon would assist architects and educators in making better decisions around the design and use of school spaces (Deppeler & Aikens, 2020; Gislason, 2010). In the following, we examine the research on designing school spaces, along with the sociomaterial intertwining of school spaces and technologies with social activities.

### How space and technologies shape school culture

School is an institution, but also a community and network of people with the fundamental goal of equipping students for the digital future (Hakkarainen et al., 2004; Korhonen et al., 2014). A school’s organizational culture guides how the school community works toward attaining goals, and it is comprised of artifacts (organizational structures and processes), shared beliefs and values, and basic underlying assumptions (Schein, 2017). We consider schools as holistic learning ecosystems consisting of an operating culture, collaboration practices and networks, pedagogic practices, digital instruments, and learning environments that involve physical, virtual, and epistemic-social environments (Nardi, 1999; Nonaka & Konno, 1998). Whereas political and educational rules, programs, and procedures, such as legislation and the national curriculum, direct structural changes, cultural change in an organization requires altering prevailing practices, habits, and assumptions (DuFour & Fullan, 2013). Hence, fostering a collaborative culture is recognized as a promising strategy for advancing teaching and learning and fostering school improvements (Casey et al., 2021; DuFour & Mattos, 2013). However, teachers’ collaboration culture varies between school communities and teachers’ informal subgroups (Meredith et al., 2017). Therefore, although teachers’ collective intention might be to participate in designing school spaces, latent tensions (e.g. rigid habits, lack of skills, power relations, and attitudes) could hinder joint efforts in generating novelty and innovation.

In addition to being an institution and a community with an operational culture, a school is also a built environment, and the design of a school building influences the school’s culture and shapes the methods of teaching and learning (Reinius et al., 2021; Stadler-Altmann, 2015). The users’ activities, in turn, reciprocally affect the further functional development of school spaces. Design solutions need to be owned by the users and supported with systems and behavioral change in order to work effectively (Higgins et al., 2005). Environments and their inhabitants have a fundamentally strong relationship; humans shape their buildings through design practice and their organizations through management practice, and the buildings reciprocally shape the activities taking place in organizations (Daniels et al., 2019). However, teachers tend to underestimate the effects of physical spaces on learning (Lei, 2010), and limited attention has been paid to materiality in learning (Fenwick, 2014) and the transition into new and more-innovative spaces (French et al., 2020). Like physical school spaces, interwoven digitalization and pedagogic practices entail systemic transformations in schools’ operational cultures and collaboration practices (Pettersson, 2021). Digitalization creates more-flexible learning opportunities (Valtonen et al., 2021) and empowers collaboration and learning on digital platforms and increases the amount of available online learning material. Digitalization and remote access, as well, enable learning and teaching that are independent of time and place, thus also improving opportunities for differentiated teaching and individual learning pathways. While old school buildings with isolated classrooms and limited digital facilities hinder achieving the full benefits of digitalization, ILEs aim to incorporate a diverse and flexible design and to favor the use of digital instruments in educational activities.

Designing school spaces requires multidisciplinary collaboration between educators, architects, IT specialists, and many other experts; educational activities and school spaces are inseparable, and users’ active participation in design is crucial for a building-in-construction to become suitable for educational activities (Daniels et al., 2019; Frelin & Grannäs, 2021; Tse et al., 2019). Although research on school buildings and environments is growing, it is limited in the educational field in Nordic countries and mostly addresses the Australian, UK, and U.S. contexts (Frelin et al., 2021). Frelin at al. (2021) identified research efforts covering the common themes of school architecture and design, policy processes, participatory design, transition to the new building, novel learning environments, and innovative or flexible learning spaces, as well as the relation between the built environment and student learning, including technology enhancement. In this study, we consider the design of ILEs as a participatory process involving the interdisciplinary community (educators, students, architects, and builders), which strives to ensure that, throughout the construction process, school spaces meet the school community’s pedagogic needs. For this process, we present a practice-oriented method to create spatial-wise knowledge about educational activities from teachers’ perspective. The method acknowledges that the collaborating non-educators understand educational activities that occur in school spaces and teachers’ expectation of the school culture, thereby consolidating the design activities of learning environments from the point of view of educational activities. Efforts have been made to create process models for co-designing school buildings (Frelin & Grannäs, 2021; Könings et al., 2017) and framing school design by combining the staff culture, student milieu, ecology, and organization aspects (Gislason, 2010). Frelin and Grannäs’s (2021) analytical model plotted stakeholders’ influence in the design and building process, in the phases of educational vision, concept design, space design, interior design, and technical design and construction. Könings et al. (2017) introduced an interdisciplinary model of practice for participatory school building design in education, illustrating how different stakeholders can participate in the process phases of plan, experiment, realize, and use. The present investigation captures teachers’ practices and perceptions of school spaces in relation to educational practices and school culture for those external to the school community to understand the school’s operational environment and culture when designing new learning environments. Next, we discuss the ontological premises of the material and social interdependence of school spaces, digital instruments, and educational activities.

### Sociomaterial approach to designing school buildings

Designing learning environments on the basis of educational activities and digital mediation entails sociomateriality. We approach the social and material aspects as equally balanced and adopt a sociomaterial approach that is conceived as post-humanist, seeking to decenter the human (Orlikowski, 2007). Fundamentally, sociomateriality interprets human and nonhuman elements as ontologically inseparable and aims to understand “the constitutive entanglement of the social and the material in everyday organizational life” (Orlikowski, 2007, p. 1435). Sociomateriality perceives the distinction between humans and artifacts only analytically (Orlikowski, 2007) and reflects Law’s (2004, p. 83) idea that “thoroughgoing relational materiality. Materials — and so realities — are treated as relational products. They do not exist in and of themselves.” In educational research, materiality tends to be separated from human subjects, considered only as background context or tools that humans use, and thus overlooking relational materiality (Decuypere & Simons, 2016; Fenwick et al., 2011). However, as Fenwick et al., (2011) highlight, materiality embodies the effects of dynamic connections and activities emerging through nonhuman and human interactions, gatherings, and everyday practices rather than simply representing discrete objects with properties. In the educational-space axis, we reflect on relational materiality as an inherent social (human interaction) and material (physical learning spaces, digital instruments, and infrastructures) entanglement. According to Decuypere and Simons (2016), relational thinking conceives of materiality in an even broader context (e.g. the district welfare or education policies of a region comprising assemblages that shape educational practices). For example, in Finland, national legislation obligates general upper-secondary schools to collaborate with higher education institutes (Finnish Act on General Upper Secondary Education, 714/2018, 8§). Thus, designing learning environments outreaches the walls of a single building, entangling digital mediation and educational activities within and beyond a school community.

School communities have a variety of (in)formal groups and collaboration practices attached to their educational activities. To expose the prevailing and emerging social phenomena related to learning environments and digitalization, we need methods capable of analyzing the individual educational activities that take place in the school spaces. Although efforts have been made to understand, for instance, social practices (Decuypere, 2020, 2021; Decuypere & Simons, 2016) and sociomaterial entanglement in empirical organizational research (Moura & Bispo, 2020), methodological discussion of the possibilities of conducting sociomaterial-based studies is limited (Moura & Bispo, 2020). In increasing the knowledge of multidimensional, sociomaterially entangled phenomena, the topological approach appears to be a promising analytical tool (e.g. Decuypere, 2021; Lamb & Ross, 2021). Regarding the presentation of sociomaterial entanglement, Decuypere and Simons (2016, p. 372) argue that “visualizations might play a crucial role” in understanding and investigating the relationality of school spaces, technology, and educational practices in the context of their use. New practice-oriented methodologies, therefore, are needed to increase understanding of the sociomaterial entanglement of pedagogy, place, people, theory, design, and practice (Carvalho & Yeoman, 2018; Cecez-Kecmanovic et al., 2014; Deppeler & Aikens, 2020) in order to design school spaces suitable for educational activities. In addressing the entanglement of digitally-mediated school spaces and educational activities in this study, we drew on relational sociomateriality (Decuypere & Simons, 2016; Fenwick et al., 2011; Moura & Bispo, 2020; Orlikowski, 2007).

### Research aims and questions

The purpose of the study was to describe how teachers perceive the needs around developing the school operational culture and their pedagogical activities in relation to school spaces. The research questions were: (1) How are school spaces, digital instruments, and teachers’ pedagogical activities entangled? (2) What kinds of socio-pedagogic educational activities should be supported and developed when designing new ILEs? (3) What kind of digital mediation do teachers perceive as supporting socio-pedagogic educational activities?

## Methods

### Research setting

The present single-case study (Yin, 2014) represents an initial phase of an ongoing longitudinal design-based research project (McKenney & Reeves, 2019) that tracing the co-design of a new school building in a life span model. In the construction process of the new building, the City of Helsinki as the municipal education provider selected the coalition of an architect studio and a private construction company in a design contest late 2020. The participatory activities with the school community were initialized rapidly after the winner was announced. A characteristic of the life span model is that the layout of the building and floor plans are highly evolved before the participatory activities. Albeit the plans are well advanced, according to the school’s socio-pedagogical needs — and within the given budget — the users and architects are still able to collaborate to alter the plans. This study was conducted a little before the design contest was closed and the participatory efforts started. Typical to empathizing and needs-based characteristics of design-based approach (McKenney & Reeves, 2019), the investigation provided practice-oriented knowledge of the school’s operational culture and spatially-entangled educational practices for the architects and construction specialists to be utilized in altering the plans according to the school’s needs. Eventually, the new building is to be deployed in 2023. The case school is a general upper-secondary school located in the Helsinki metropolitan area and with around 50 subject teachers. The current number of the approximately 650 students is expected to increase of 200 when the new building is deployed. In addition to regular general upper-secondary school studies, the school offers advanced subject-specific training. The current school building was constructed in the 1960s, with traditional corridors and closed classrooms comprising the school spaces. Large school communities are typical in the metropolitan area and, although the municipal education providers have put a lot of effort in renewing school facilities, many schools are waiting to renew their own.

To integrate the understanding of educational practices early into the construction process, this investigation focused on exposing the teachers’ perspective. The study relied on a research-practice partnership (RPP), which is considered as a promising approach in improving educational practices (Coburn & Penuel, 2016). The RPP aimed at engaging teachers in the construction process of the new school building with multiple stakeholders, such as a municipal education provider (organizer of the new building design contest, and building owner) and a university (a research party). The RPP community also included architects, interior designers, and constructors of the service provider (winner of the design contest), but they were omitted from this study because the design contest process was unfinished by the time the data were collected.

### Data acquisition

The present mixed-methods research relied on the triangulation of data collected in fall 2020 (Table 1). We organized a workshop for the teachers (*n* = 39) of the present schooland conducted a teacher survey (*n* = 31) and semi-structured interviews (*n* = 10).

**Table 1:** Summary of data, participants, and analyses

Data	Participants	Analysis
Teachers’ Activities and School Spaces Inventory (TASSI) as an online survey (Qualtrics): close-ended items (1–7 Likert evaluations) and open-ended items for verbatim reporting by choice	Teachers, *n* = 31	Quantitative content analysis (Excel)
Collective digital platform (Padlet) outcome (.pdf) from the workshop	Teachers, *n* = 39	Qualitative content analysis (Atlas.ti)
Semi-structured interviews (total 11 h 37 min, length varied between 46–81 min)	Teachers, *n* = 10	Qualitative content analysis (Atlas.ti)

To address research question 1, we administered the Teachers’ Activities and School Spaces Inventory (TASSI) during an official teacher meeting. The questionnaire contained the responses of 31 volunteer teachers with informed consent, who represented the views of almost all the full-time teachers and accounted for 62% of all 50 teachers in the school. The inventory was intended to trace the sociomaterial entanglement of teachers’ socio-pedagogic activities and their ways of using school spaces. Toward that end, we identified teachers’ main socio-pedagogic activities and their subordinate dimensions in relation to their ways of using the school spaces. To support the data collection, we conducted a qualitative content analysis of the school’s pedagogic plan, national curriculum, and the architectural requirements for the new building. We inductively identified eight preliminary educational categories and 59 subcategories attached to educational renewal, and found 216 connections to educational activities from the architectural requirements. Iterative grouping and condensing resulted in six socio-pedagogic thematic categories, each including four to nine descriptive subcategories. The six main categories structuring the questionnaire and interviews were: (1) community and communal learning, (2) learner wellbeing and inclusion, (3) future transversal competencies, (4) ubiquitous learning, (5) digital design of physical learning environments, and (6) design of virtual environments. In the context of each category, we determined how the aspects of school spaces relate to (a) the school’s operating culture, (b) teachers’ pedagogical goals, and (c) teachers’ practices of using school spaces (see Table 2 for an example).

**Table 2:** Example of implemented claims and culture, goals, and usage evaluation scales in TASSI

Socio-pedagogic activity		Spaces from point of view of school’s socio-pedagogic activities		
		*Claim on spaces related to activities of school’s operating culture overall*	*Claim on spaces related to teacher’s own pedagogical activities*	
*Category*	*Subordinate*	*(a) Evaluation: Importance from the perspective of the overall school culture (1 = Not at all important – 7 = Very important)*	*(b) Evaluation: Importance from the perspective of achieving teacher’s own pedagogic goals (1 = Not at all important – 7 = Very important)*	*(c) Evaluation: How often was the statement true (usage) during the last school year (1 = never, 2 = few times a year, 3 = once a month, 4 = few times per month, 5 = once a week, 6 = few times per week, 7 = daily)*
Community and communal learning	Communal knowledge building and sharing	The new premises have an open and easily accessible flexible multifunctional space that encourages doing and experimenting together.	In my teaching, I make versatile use of the school’s open multifunctional spaces as a learning environment.	

We implemented the TASSI as a Qualtrics online questionnaire, with teachers evaluating the claims on a 1–7 Likert scale from three perspectives: importance (1 = not at all important to 7 = very important) from the perspective of the overall school’s *culture* (Table 2a), importance in achieving the teacher’s own pedagogic *goals* (Table 2b), and intensity of *usage* (1 = never, 2 = few times a year, 3 = once a month, 4 = a few times a month, 5 = once a week, 6 = a few times a week, and 7 = daily) (Table 2c). When responding, the teachers were asked to consider the last school year. Regarding digital mediation-related claims, we inquired only about teachers’ perceptions of the overall importance of digital instruments, rather than inquiring separately about their importance in terms of the school’s operating *culture* and in achieving pedagogic *goals*. Although this omission was methodologically inconsequential, it limited the information obtained from digital instruments to two dimensions (i.e. *usage* and *overall importance* of digital mediation, and lacking the *goals* and *culture* perspectives). In addition to the claims, the questionnaire included open-ended items for the teachers to report their expectations around new school spaces verbatim. Hence, the TASSI inventory was designed to trace the teachers’ perceptions of the entanglement of learning spaces and pedagogic practices rather than to assess these aspects separately.

To answer research questions 2 and 3, we enriched the survey data by organizing a workshop for teachers (*n* = 39) and conducting semi-structured teacher interviews (*n* = 10). The workshop was organized during an official teacher meeting. Participating in the workshop was voluntary. We designed the workshop in collaboration with the school principal, a group of teachers (*n* = 5) who were assigned to the new school building construction team representatives, an education specialist, and an architect of the municipal education provider. As to the TASSI, the aim of the workshop was to collect data about teachers’ perceptions of school spaces and educational activities entanglement, which were to be shared with the stakeholders involved in the design and construction of the forthcoming building. Because of the Covid-19 pandemic, the workshop was organized in a spacious hall with safe distances. In the workshop, we challenged teachers to consider spatial dimensions in educational renewal. First, during a few introductory remarks, one architect shortly presented a few general pictures of collaboration premises. Thereafter, we asked teachers to work in groups of three to four and write down on a collective digital platform (Padlet) what they found important in benchmarking ILEs. The teacher groups dispersed to separate spaces and contributed to the collaborative Padlet platform. The Padlet was organized according to four thematic headings closely corresponding to the TASSI themes (community and communal learning, future skills, learner wellbeing and inclusion, ubiquitous learning, and digital instruments as enablers of creative learning and teaching). An additional column was reserved for mind-boggling topics around school spaces. Thereafter, the groups presented the contributions recorded on the Padlet in the shared space, while we took field notes. Further, we performed and audio recorded semi-structured interviews with the teachers according to the nature of their TASSI responses. The interviewees represented various subject domains and digital-collaboration profiles; three were men and seven were women. The interviews aimed to investigate teachers’ perceptions of teacher collaboration, the learning and teaching of futures skills, and their views of digital mediation and school spaces design in relation to educational renewal.

### Data analysis

**Fig. 1:**
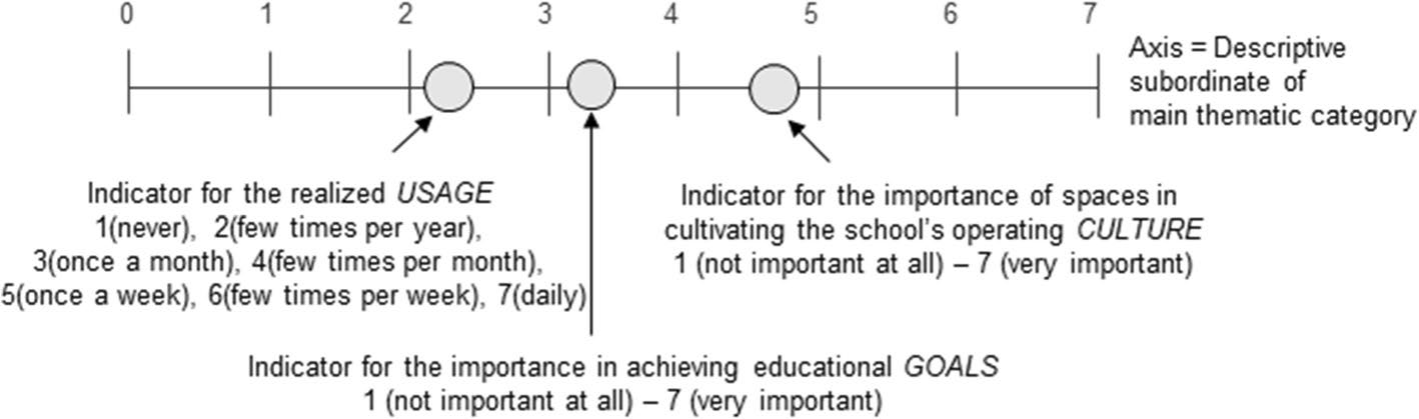
Tenets of calculated indicators plotted into a socio-pedagogic thematic axis

To explore the entanglement of school spaces, digital instruments, and teachers’ pedagogical activities (research question 1), we developed a methodological framework for tracing sociomaterial entanglement^1^. The acquired quantitative TASSI data (i.e. Likert evaluations) were organized in Excel according to the thematic main categories and subcategories. For each subcategory-related questionnaire item, we calculated three means, namely, importance in relation to school *culture*, teachers’ pedagogic *goals*, and space *usage* indicators, corresponding to the three perspectives by which teachers had evaluated the claims. The data analysis involved creating six radar charts, one for each main thematic category. We set the descriptive subordinates as the axes, on which we plotted the related *culture*, *goals*, and *usage* indicators (Fig. 1). In each radar chart, each axis had three plotted indicators, except for the two digital mediation-related charts which had only two indicators because of the omission in data acquisition. To expose the educational practices and digital mediation that should be supported and developed when designing new ILEs (research questions 2 and 3), we identified two types of gaps from the created radar charts. We looked for subcategories whose *culture* and *goals* indicators differed the most from each other, thus exposing practices that the teachers perceived as important but not currently being realized. We also looked for subcategories in which the *culture* and *goals* difference to the *usage* indicator was greater, thus exposing activities that the teachers perceive as important though unsupported in the current spaces.

**Fig. 2:**
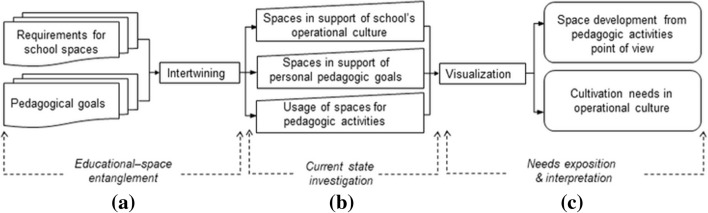
Methodological framework for tracing sociomaterial entanglement

Our analysis focused on how school spaces, digital instruments, and teachers’ pedagogical activities are entangled. We developed a methodological framework for examining the entanglement of teachers’ socio-pedagogic activities and school spaces through three phases (Fig. 2).

The *educational-space entanglement* phase (Fig. 2a), assisted in identifying socio-pedagogic activities and their manifestation in school spaces, connecting the educational goals of the school, municipal education provider, and national curriculum, as well as the architectural requirements for the new school building.

The *current state investigation* phase (Fig. 2b) helped to obtain information about teachers’ perceptions of school spaces and educational activities (e.g. Likert scale evaluation on claims attached to the socio-pedagogic activities) from three perspectives:

- *Culture* interpreted importance of spaces in cultivating operational culture.
- *Goals* perceived importance of spaces in achieving teachers’ own pedagogical goals.
- *Usage* experienced use of spaces.

The *needs exposition and interpretation* phase (Fig. 2c), involved visualizing the obtained information for human interpretation. First, means were calculated for each socio-pedagogic activity for all three perspectives serving as the *culture*, *goals*, and *usage* indicators for the activity. *Charts* were created for human interpretation by plotting the means to axes representing the evaluated socio-pedagogic activities. Finally, differences in the *culture* and *goals* indicators within the thematic axes were investigated to expose the needs in terms of cultivating the school’s operational culture. Substantial differences between the *usage, culture*, and *goals* indicators were investigated to identify the needs in designing the school spaces to support the socio-pedagogic activities. Investigating the differences also aimed to identify the development needs in terms of the school’s operational culture.

To evaluate our heuristic findings from the charts visualizing the quantitative TASSI data, we conducted qualitative content analysis of the workshop and interview data in Atlas.ti (Friese, 2012; Saldãna, 2013). We used one mention or an idea comprising the smallest coherent unit of meaning as the unit of analysis and applied descriptive coding. First, we familiarized ourselves with the workshop data by reading the collective digital platform (i.e. Padlet) outcome. We coded the data according to the subcategories and grouped the resulting 115 coding occurrences according to the overarching main categories. Next, we transcribed the interviews word by word and anonymized them for qualitative content analysis. We again familiarized ourselves with the data by reading the transcriptions and partitioned the data into 54 text segments related to school spaces and 30 to digital mediation. We inductively coded the segments in relation to the research questions. In the results section, the depicted data excerpts are answers to the open-ended items of TASSI, as translated by us. We edited the excerpts minimally to promote indigenousness and marked deletions with three dots […].

## Results

In this section, we report our findings of the analysis around designing ILEs that support educational practices and digital mediation. Firstly, we examine the kinds of socio-pedagogic activities that should be supported when designing new ILEs. Secondly, we examine the digital mediation methods that teachers considered relevant in fostering the socio-pedagogic activities. Finally, we summarize the results.

## What kinds of socio-pedagogic educational activities should be supported and developed when designing new ILEs?

Below, we present the results across four main themes of socio-pedagogic activity: (1) community and communal learning, (2) wellbeing and inclusion, (3) future transversal competencies, and (4) ubiquitous learning. The results rely on both quantitative and qualitative data.

### Collaborative activities

**Fig. 3:**
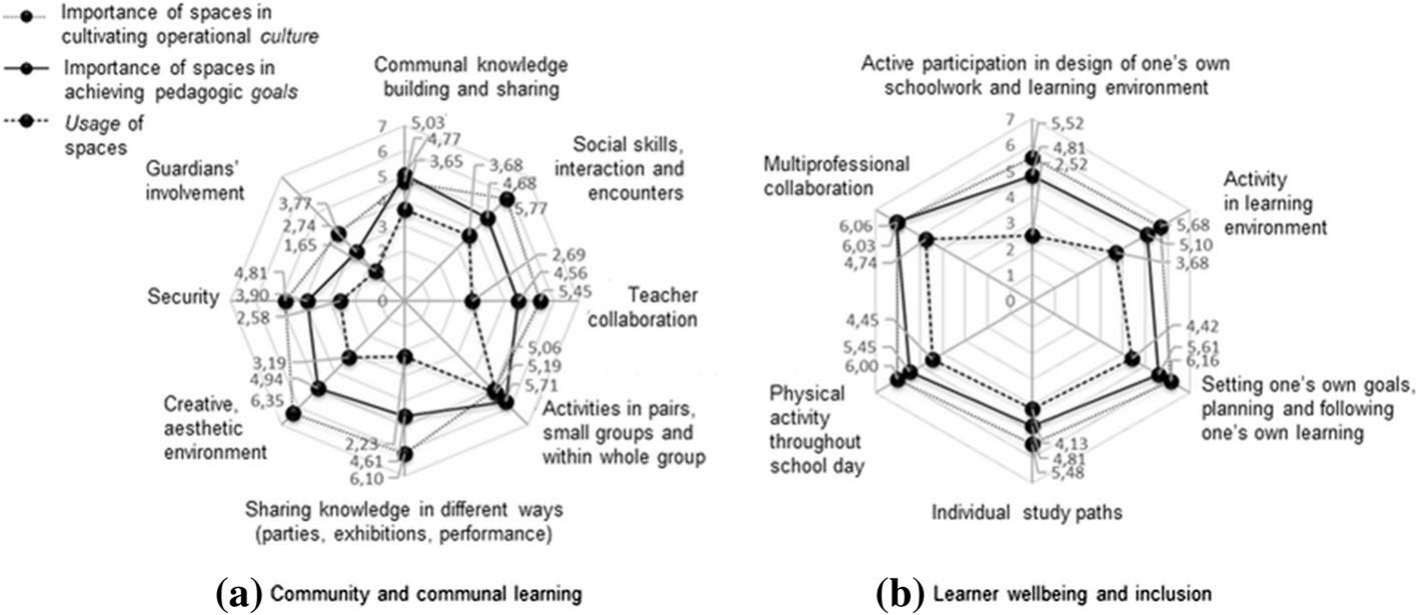
Community and communal learning, learner wellbeing and inclusion

The radar chart in Fig. 3a that visualizes educational activities of collaborative characteristics reveals that teachers considered it vital to cultivate collaborative and communal sentiments. Collaboration covered many highly-emphasized themes, such as teacher collaboration, social interaction, communal knowledge building, and multiprofessional collaboration (Fig. 3b). The workshop data reflected the need to cultivate community and communal learning, including teacher collaboration. From the interviews, the main finding was the need for diverse and flexible spaces that would accommodate bilateral meetings, students’ teamwork and large-scale communal events. The interviewed teachers also called for spaces supporting collegial collaboration in planning and teaching, as separate from the staffroom that was intended for bonding and the spaces designed for concentration-intensive work. However, participants did not raise the importance of collaboration with guardians; generally upper-secondary school students were presumably expected to function in a self-directed way. Although the novel core curriculum emphasizes teacher collaboration in the educational activities that ILEs aim to foster, teachers reported contradictory feelings on teacher collaboration:

“Our school has a well-established sense of working alone.” (Teacher A).

“Co-teaching where two teachers guide a huge group of students doesn’t work, and I don’t get excited about it.” (Teacher B).

“It is easier to manage large teaching groups with more teachers.” (Teacher C).

The interviewed teachers expressed only a few thoughts related to aesthetics or interior design matters, such as furniture and natural light, probably because the collaborative activities of the new school design were in the beginning stages. Only one comment in the interviews related to security, namely, considering open spaces as a risk when there is a need for sheltering in threatening situations. Some teachers preferred closed classrooms instead of increased openness in the forthcoming teaching spaces:

“I wish there were classes as before.” (Teacher D).

“In a room isolated with glass walls or partitions, I don’t want to teach.” (Teacher E).

### Learner wellbeing and inclusion

Community and collaboration-related findings emphasized the importance of learners’ wellbeing and inclusion in school spaces (Fig. 3b); wellbeing is an important emphasis in the curriculum as well. Additionally, workshop data related to student guidance, multiprofessional student welfare group activities, and students’ holistic wellbeing. The participants, however, did not address learners’ wellbeing in relation to school space design in their interviews. Although student guidance and individual needs were considered important to take into account in planning the new building, according to the survey data, teachers perceived student counseling activities as cumbersome:

“The teaching groups are so large that individual counseling is almost impossible.” (Teacher F).

Teachers perceived the current spaces as generally supporting learners’ wellbeing and inclusion relatively well. The spaces, in contrast, did not support learners’ active participation in the design of their own schoolwork and learning environment. One teacher encapsulated the expectations around school spaces supporting learners’ activity, wellbeing, and inclusion as follows:

“For students, I would like not only ping-pong-like activities, but also plenty of flexible seating groups that would really be accessible to everyone (not just alpha males and females) and flexible small group spaces for work.” (Teacher A).

### Cross-curricular and interorganizational culture

**Fig. 4:**
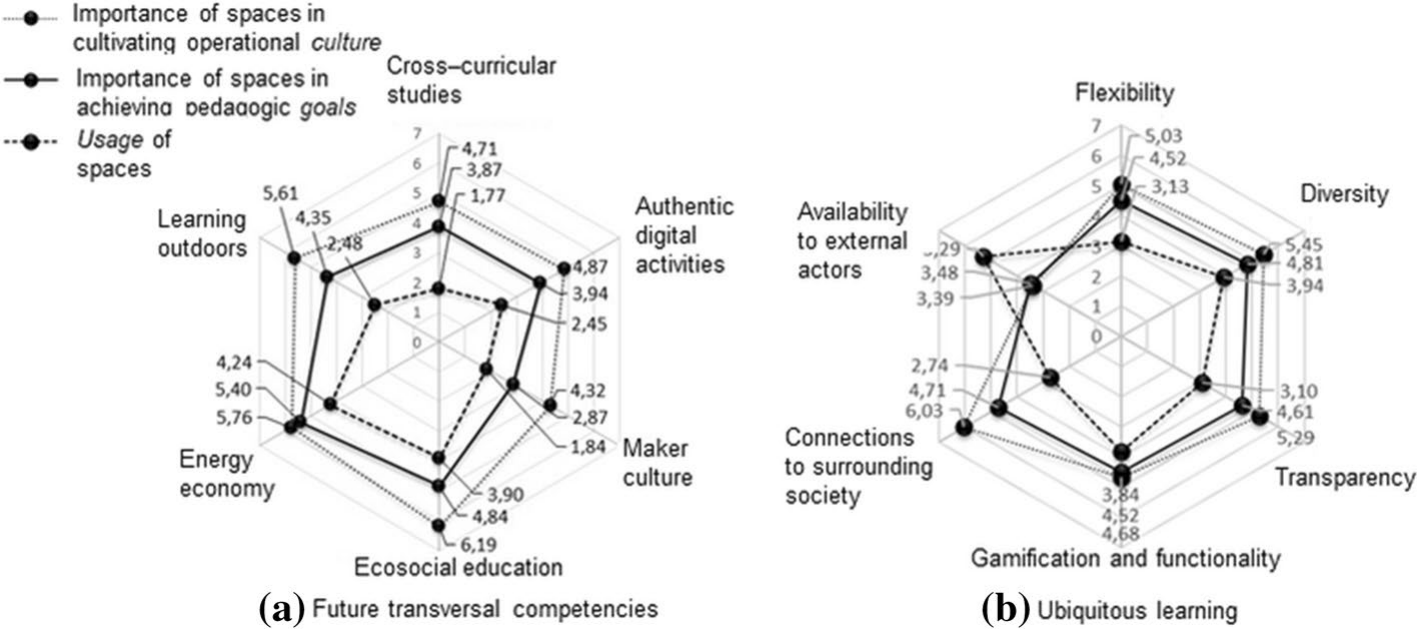
Future transversal competencies and ubiquitous learning

We examined future transversal competencies and ubiquitous learning that were related to the integration of subject domains and open schools in society by building links between working life and higher education. In addition to cross-curricular activities, general upper-secondary schools in Finland are obligated to collaborate with higher educational institutes and companies. The teachers evaluated cross-curricular studies as quite important but rarely realized in the current premises (Fig. 4a). The interviewed teachers reported the need for spaces to be well-suited to collaborative planning and the combining of teaching groups, although interest in such collaboration varied. One survey response summarized the variation in the teachers’ interest in intra- and inter-organizational collaboration as follows:

“Teachers who are interested in the development and design of joint courses are always the same. The actual implementation of the LOPS [i.e. the new national curriculum for general upper-secondary schools] would require an active interest from all.” (Teacher K).

In accordance with the findings, the workshop data consisted mostly of subject or individual study path orientations, but they lacked the notion of cross-curricular studies and activities supporting a collaborative culture. School spaces did not provide sufficient support for authentic and versatile learning activities, such as having virtual meetings with external partners and authentically simulated cultural practices with virtual reality (VR) or augmented reality (AR). Figure 4a also brought up how important it is to design spaces to support maker culture and utilize outdoor spaces as learning environments. Further, it was examined how the values of a sustainable future (e.g. energy economy and ecosocial education) were present in the design of ILEs and pedagogic activities.

The teachers evaluated connections to the surrounding society (Fig. 4b), such as two-way company visits, as vital to schools. During the last school year, 23 out of 31 teachers had participated in the development of inter-organizational collaboration, either with educational institutes or companies. Company collaboration, however, was currently rare, with school spaces lacking support for external visitors. Half of the interviewed teachers discussed inter-organizational collaboration and interlinked it to flexible spaces enabling larger school community gatherings. Further, the interviewed teachers considered visits to external premises as being more authentic learning experiences than events organized at school spaces, although the survey data exposed this as challenging because of a lack of suitable facilities in companies:

“It is difficult to take groups of more than 30 students to companies […] companies often do not have such facilities. Yes, we do get visitors to schools, but it would be a more authentic experience for students to visit companies.” (Teacher G).

All the data revealed that, in designing learning environments, flexibility, diversity, and transparency supporting openness in knowledge sharing are frequently needed in daily educational activities (Fig. 4b). The workshop and interview data, however, revealed acoustics as a design concern, and teachers considered the soundproofing of the adjustable partitions as inadequate (e.g. in teaching languages or music). Nevertheless, the consideration of sustainability in school spaces was at a relatively low level; the workshop and interviews did not contain references to these issues. The new school building, however, provides an example of diverse waste-sorting opportunities, which is commonplace in Finland. The case school is also part of the world’s largest sustainable schools Eco-School program. Lastly, the chart (Fig. 4b) reveals that school spaces are available to an adult high school operating in the case school premises but, because this was considered unimportant from the perspective of the school’s daily activities, it was not mentioned in the workshop and interview data.

## What kinds of digital mediation do teachers perceive as supporting socio-pedagogic educational activities when designing new ILEs?

In this section, we examine the *overall importance* of using various aspects of digital technologies in cultivating the school’s operational culture and achieving educational goals, as well as how often these principles were realized (i.e. *usage*) in school spaces.

### *Parallel design of readily available digital instruments with inservice support.*

**Fig. 5:**
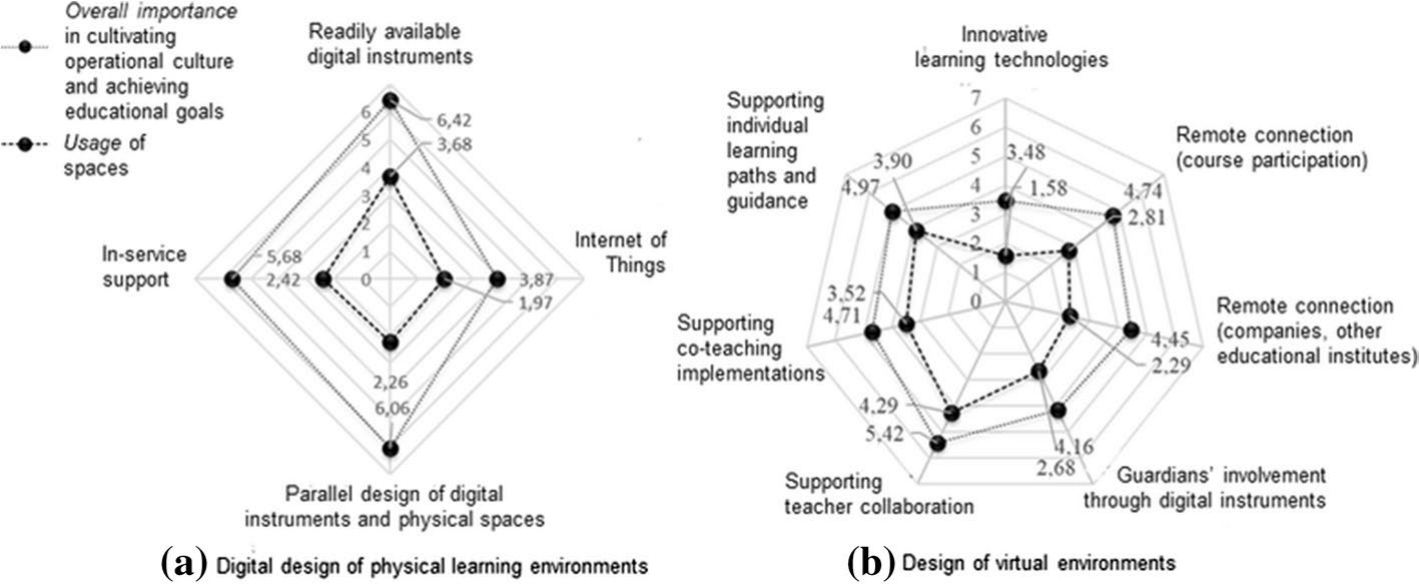
Digital design of physical learning environments and design of virtual environments

Overall, teachers considered digital instruments to be highly important in educational activities (Fig. 5). They considered it important to have digital instruments and practical support readily available, as well as for digital instruments to seamlessly integrate into the learning spaces (Fig. 5a), albeit this was currently inadequately realized. However, the teachers evaluated innovative technologies, such as immersive technologies, VR, AR, and objects embedded with sensors (IoT, Internet of Things), in school spaces as being unimportant and rarely used. Presumably, many teachers were unfamiliar with innovative technologies and their related benefits in teaching. Echoing these findings, the expectations of all interviewees toward digital instruments in pedagogic use were practical, and the workshop data findings prioritized functional and effortlessly accessible digital instruments; teachers essentially prioritized basic level functionality before novel technological solutions in the learning environments:

“That the basics work before you show off with immersions and sound showers, e.g. enough plugs and extension cords, and a working internet.” (Teacher H).

Additionally, the interviewed teachers frequently reported the need for multiple reflection possibilities in the learning spaces. Teachers attached the diverse reflection possibilities to students’ ergonomics as well as differentiated learning tasks (e.g. slower progressing and more advanced students).

Echoing the above findings, the examination of virtual environments (Fig. 5b) revealed that digital instruments were considered to be important in intra- and inter-organizational collaboration, as well as in support of individual learning paths that the core curriculum emphasizes. One teacher related these instruments to inclusion and educational activities and crystallized the goal of digitalization in learning environments as follows:

“Virtual learning environments should enable students to experience inclusion and be able to participate fully in group activities through them.” (Teacher I).

However, teachers considered that the role of innovative learning technologies was insignificant and that they were currently rarely used. They were concerned about the dysfunctionality of digital instruments, especially in relation to remote connections. Further, the interviewed teachers called for adequate audiovisual remote connections to external stakeholders, such as companies and universities. In regard to digital means, teachers assessed guardians’ involvement as quite important, although not currently realized in practice; the role of guardians, however, was not addressed in the workshop and interview data. Overall, we found only a few separate mentions of digital instruments and virtual environments in the workshop data. The teachers considered usage of innovative technologies, such as VR, AR, and immersive learning environments, as being insignificant and absent in the data:

“I don’t know what kind of materials will come into the new curriculum again, but the level of finesse of the virtual material depends on it. Personally, I do not work with these.” (Teacher A).

“I trust the vision of the designers.” (Teacher J).

“Virtual learning environments feel somehow secondary at the moment; with almost 40 students in groups, it is mainly mass management.” (Teacher G).

To summarize, the analysis revealed certain main themes that emerged in the analysis across the three bodies of radar charts and workshop and interviews data (Table 3). The findings resonate with the Finnish general upper-secondary schools’ educational goals of increasing versatile collaboration and holistic wellbeing, as well as usage of digital instruments in teaching and learning (EDUFI, 2019).

**Table 3:** Main themes revealed through the results analysis

Radar charts	Workshop	Interviews
Collaborative activities	Cultivation of community and communal learning, including teacher collaboration (35 mentions) Flexibility and diversity of spaces, and transparency supporting openness in knowledge sharing (24 mentions)	Teacher collaboration and community building (29 quotations). Diversity and flexibility of spaces (39 quotations).
Learners’ wellbeing and inclusion	Multiprofessional student welfare group activities, student guidance, and students’ holistic wellbeing (27 mentions)	No related quotations.
Cross-curricular and interorganizational culture	Connections to surrounding society (11 mentions); no mentions of cross-curricular activities	Cross-curricular teacher collaboration (4 quotations) Interorganizational collaboration (10 quotations)
Parallel design of readily available digital instruments with inservice support	Functional and effortlessly accessible digital instruments (13 mentions)	Basic-level functionality prioritized before novel technological solutions (20 quotations)

## Discussion

The purpose of this study was to describe how teachers perceive the needs around developing the operational culture of schools and their pedagogical activities in relation to school spaces. The study was conducted to feed teachers’ practice-oriented knowledge to the initial phase of a longitudinal design, construction, and deployment process of a new school building conducted in RPP. The first research question concerned how school spaces, digital instruments, and teachers’ pedagogical activities are entangled. The developed methodological framework for tracing the entanglement of teachers’ socio-pedagogic activities and school spaces responded to the call for methodological discussion on sociomaterial studies (Moura & Bispo, 2020) and their practical relevance (Cecez-Kecmanovic et al., 2014). Although the design and layout of the new case school building were advanced because of a design contest, the life span model enables collaborative efforts in altering the learning environments according to the school’s educational needs. The participatory efforts that are parallel to the construction entail understanding of the educational activities occurring in school spaces for the multidisciplinary stakeholders.

While visualization is central in understanding and investigating context-dependent and digitally-infused learning environments and educational practices (Decuypere & Simons, 2016), the novel method integrated qualitative and quantitative approaches to transform teachers’ subjective perceptions on socio-pedagogic activities that occur in learning environments to quantified visualizations for human interpretation. The practice-oriented and collaborative RPP research setting in the longitudinal construction process of a contemporary school building since the beginning provided a fruitful context for understanding practitioners’ perceptions of educational foundations in creating learning environments for the future. The knowledge generated increases multidisciplinary stakeholders’ understanding of the educational practices occurring in school spaces and, thus, consolidates the design of ILEs to meet pedagogic needs. The framework provided analytical tools and visualizations for tracing entanglement in the use of educational practices, school spaces, technology, and practices to be utilized in an multidisciplinary co-design (Carvalho & Yeoman, 2018; Decuypere & Simons, 2016).

Using rather simple mathematics, radar charts with the three indicators provided a deeper understanding of relational sociomateriality within educational practices and were suitable for human interpretation. The framework provided tentative clarity for operationalizing sociomaterially-entangled educational practices in the school design process (Daniels et al., 2019). It promoted a deeper understanding of the mutual shaping involved in the design of school spaces and the related pedagogical renewal (Daniels et al., 2019; Frelin & Grannäs, 2021; Gislason, 2010; Könings et al., 2017). The forthcoming school building needs to provide learning environments where teachers are able to execute the educational goals of the recently-introduced core curriculum. The teachers’ perceptions and practices, for example, on cross-curricular activities and inter-organizational collaboration, varied as well as subject-specific teaching needs. Making these varying needs explicit in an early phase to a construction process strengthens the non-educators’ understanding of the school’s daily educational activities, habits, and obligations. According to our expectations, the use of the case school’s current spaces to support collaborative and digitally-mediated educational activities remained rather limited because of the design of the old school building. Therefore, the current facilities are not capable of supporting educational activities in line with the new curriculum and digitalization. The interpreted data sets provide a fruitful reference point for tracing how sociomaterial relations transform in the process of constructing new school buildings and renewing the operational culture of schools.

The second question led us to investigate what kind of socio-pedagogic educational activities should be supported and developed when designing new ILEs. Supporting earlier findings on the importance enhancing collaboration in schools (Casey et al., 2021; DuFour & Mattos, 2013), our results indicated that it is critical to design ILEs to strengthen collaborative practices around cross-curricular and cross-organizational activities. The findings also echo the new national curriculum’s recommendation of teacher collaboration (EDUFI, 2019). For instance, the municipal education provider requires that all general upper-secondary schools organize a sustainable future-related theme course. The course consists of cross-curricular study modules and is compulsory for all first-year students; thus, teacher collaboration in different subject domains is required. The diversity and flexibility of spaces that the teachers in this study called for appear to enable such collaboration and heterogeneous learning groups. In addition to the diversity of learning environments, teacher collaboration and cross-curricular courses entail the need for spaces that allow joint planning by teachers, in addition to those that allow for teachers’ natural bonding and interaction in generating new types of collaboration. The diverse and flexible spaces that the teachers emphasized are needed to enable individual study paths, educational inclusion, and differentiated learning tasks. Teachers were concerned about glass walls and the acoustic features of learning environments in relation to focused individual study, small-group activity, and students’ stage fright when others see them giving presentations. The teachers’ emphasis on the need for flexible spaces reflects the increased variation in group sizes in schools, along with the increase in the number of students per school, which is commonplace in Finland today.

The third question addressed the kinds of digital mediation teachers that perceive as supporting socio-pedagogic educational activities. Our results suggest that school spaces and digital instruments should be designed in parallel, including support for using technologies in practice. From the perspective of meeting educational goals, vital teacher collaboration entails national curricular objectives that aim to equip students with the skills that educators perceive as important for the future (Hakkarainen et al., 2004). Although the skills that will actually be needed in the future are only best guesses, digitally-mediated, interdisciplinary collaboration probably will be in demand. In this regard, the teachers in this study perceived the need for adequate digital instruments, both in onsite and remote learning, reflecting the need for schools to provide a contemporary learning environment. Digitally-mediated ILEs enable schools to enhance individuality in learning and skills attainment and, further, they indirectly support the ultimate goal of educating versatile professionals for the future society. However, teachers perceived advanced VR and AR as secondary. In this regard, the findings indicate that innovative learning technologies need to mature and partnerships need to be created between developers, researchers, and teachers in order to develop workable ways of utilizing them in schools.

### Limitations and further considerations

The study presented empirical RPP data concerning teachers’ envisioning of the school’s operational culture and pedagogic goals in relation to school spaces for use in the interdisciplinary efforts in constructing a school building. Although the case school represents the typical size of a general upper-secondary school in the Helsinki metropolitan area, one of the limitations was the small sample size involved in studying only one school and a relatively-small number of participants. Regarding reliability and validity, a different sample might have delivered different conclusions. To promote the integrity and alliance of data and analysis, we relied on the mixed-methods approach for tracing teachers’ perceptions of educational practices and their expectations around the creation of new spaces. We contributed to the methodological discussion and introduced a framework for operationalizing the entanglement of school spaces and educational activities in the digital era. The framework provided a visualized sociomaterial interdependence for human interpretation. Operationalization of the created framework also revealed where perceptions of the importance of spatial-wise socio-pedagogic activities collide with real-life counterparts, such as guardians’ collaboration required by the law (Finnish act on general upper secondary education, 714/2018, 31§) and the perceived importance of related activities in the case school.

Our RPP design-based research approach appeared to reflect a sociomateriality that was applicable to the interdisciplinary stakeholders involved, namely, educators and architects. Both educational design research (McKenney & Reeves, 2019) and RPP (Coburn & Penuel, 2016) aim to improve educational practices. The practical implications of the investigation derived from redirecting the findings to the RPP community. The case school principal gained knowledge around cultivating educational practices and promoting teachers’ professional development in new school spaces, thus adhering to the priority of national educational renewal. Consequently, professional training was organized on the themes identified as important but unrealized. These were co-teaching and encountering a multicultural environment to cultivate collaborative practices and wellbeing. Training on innovative digital technologies in promoting learning will be organized in the forthcoming academic year. The interdisciplinary group collaborating toward the new school also led to new knowledge about the relevant practices pertaining to the provision of contemporary school spaces and educational renewal.

The introduced method was developed early when the construction process of the new building and participatory efforts with the school community were about to begin. The quantified sociomaterially entangled indicators assisted tracing teachers’ perception on educational activities in relation to school spaces. It is anticipated that the method will evolve along with the longitudinal RPP design-based research project collaboration. When collecting the TASSI data after the new school building is deployed, comparing the mean indicators in the created visualizations enables tracing of the educational transformation within the case school. However, when using TASSI to acquire data in other contexts, it is critical to ensure that wording of items reflects school-specific activities. Another challenge is to specify the *culture*, *goals*, and *usage* claims to measure the same main theme within one subordinate.

## Conclusion

To conclude, we argued for a vital need to consider teachers’ perspective to the entanglement of school spaces, digital instruments, and pedagogic practices when designing and constructing new school buildings from educational goals point of view. Our findings support the interdisciplinary design of innovative digital instruments from the perspective of socio-pedagogic activities, thus ensuring a simultaneously adequate operational reliability. The present findings contribute to understanding of the interrelations between school spaces, mediating digital instruments, and teachers’ socio-pedagogic activities.

## Data Availability

Not applicable.

## Footnotes

1 For the TASSI inventory and the mathematical details for implementing the methodological framework, please visit our website (https://growingmind.fi/sociomaterialmethod/).

## Code availability

Mathematical details and the TASSI survey for implementing the methodological framework are available on our website: https://growingmind.fi/sociomaterialmethod/.
